# Changes in Bone Mineral Density and Serum Lipids across the First Postpartum Year: Effect of Aerobic Fitness and Physical Activity

**DOI:** 10.3390/nu14030703

**Published:** 2022-02-08

**Authors:** Erin M. Kyle, Hayley B. Miller, Jessica Schueler, Michelle Clinton, Brenda M. Alexander, Ann Marie Hart, D. Enette Larson-Meyer

**Affiliations:** 1 Department of Family and Consumer Sciences, University of Wyoming, Laramie, WY 82071, USA; erin.kyle.728@gmail.com (E.M.K.); jesschueler@gmail.com (J.S.); michelle_felts@live.com (M.C.); 2 Department of Human Nutrition, Foods and Exercise, Virginia Tech, Blacksburg, VA 24061, USA; hayley@vt.edu; 3 Department of Animal Science, University of Wyoming, Laramie, WY 82071, USA; balex@uwyo.edu; 4 School of Nursing, University of Wyoming, Laramie, WY 82071, USA; annmhart@uwyo.edu.com

**Keywords:** bone density, bone resorption markers, HDL-C, LDL-C, lipid profile, cardiovascular disease, exercise, aerobic fitness

## Abstract

This study evaluated the changes in bone mineral density (BMD) and serum lipids across the first postpartum year in lactating women compared to never-pregnant controls, and the influence of physical activity (PA). The study also explored whether N-telopeptides, pyridinoline, and deoxypyridinoline in urine serve as biomarkers of bone resorption. A cohort of 18 initially lactating postpartum women and 16 never pregnant controls were studied. BMD (dual energy X-ray absorptiometry), serum lipid profiles, and PA (Baecke PA Questionnaire) were assessed at baseline (4–6 weeks postpartum), 6 months, and 12 months. Postpartum women lost 5.2 ± 1.4 kg body weight and BMD decreased by 1.4% and 3.1% in the total body and dual-femur, respectively. Furthermore, BMDdid not show signs of rebound. Lipid profiles improved, with increases in high-density lipoprotein-cholesterol (HDL-C) and decreases in low-density lipoprotein cholesterol (LDL-C) and the cholesterol/HDL-C ratio at 12 months (vs. baseline). These changes were not influenced by lactation, but the fall the Cholesterol/HDL-C ratio was influenced by leisure-time (*p* = 0.051, time X group) and sport (*p* = 0.028, time effect) PA. The decrease in BMD from baseline to 12 months in total body and dual femur, however, was greater in those who continued to breastfeed for a full year compared to those who stopped at close to 6 months. Urinary markers of bone resorption, measured in a subset of participants, reflect BMD loss, particularly in the dual-femur, and may reflect changes bone resorption before observed changes in BMD. Results provide support that habitual postpartum PA may favorably influence changes in serum lipids but not necessarily BMD. The benefit of exercise and use of urinary biomarkers of bone deserves further exploration.

## 1. Introduction

Pregnancy and lactation are characterized by a variety of physiological changes in the mother, including changes in bone mineral density (BMD) and lipid profiles. Extensive research has found significant decreases in BMD [1,2,3] and increases in plasma cholesterol and triglyceride concentrations throughout the gestational period [4,5,6]. While maternal adaptation, including increased absorption of intestinal calcium [7] and changes in lipid metabolism [8], help ensure supply of calcium and lipid to the fetus and placenta for steroid hormone synthesis during pregnancy, the persistence of reduced BMD and elevated serum lipids and triglycerides (TG) during the postpartum period can become risk factors for lactation-induced osteoporosis [9] or cardiovascular disease [10].

According to the Institute of Medicine, lactating women provide two-to-three times more calcium to their infant through breast milk during the first 6 months of breastfeeding than during the entirety of pregnancy [11]; this results in significant alterations in maternal calcium metabolism to maintain serum calcium within the normal range [7,12]. While increased intestinal calcium absorption helps accommodate these demands during pregnancy, the primary mechanisms of calcium conservation during lactation include renal resorption via the distal tubules, skeletal demineralization, and bone resorption [7]. Additionally, studies have found that neither calcium intake nor supplementation influences lactation-induced BMD loss [13,14,15], highlighting that these changes are physiologically driven. Previous research has found inconsistent results regarding the impact of lactation on BMD loss and its subsequent recovery that may vary by skeletal site and length of lactation. Several studies observed significant decreases in BMD from baseline in women lactating at least 3 to 6 months [1,2,3,16,17,18,19] with those who breastfed more than 4 to 6 months experiencing greater bone loss than those that weaned earlier [1,2,3,16,20,21]. In longitudinal studies with mostly young, healthy, Caucasian mothers, BMD was shown to decrease ~2 to 7.5% during the first 4–6 months of lactation in the lumbar spine and/or hip [2,3,13,16,18,19,20,22,23,24], 0 to 5% in the forearm radius [13,16,19,22,24], and 0 to 3% of the total body BMD [2,3,13,18,19,24]. Although most BMD loss within the first 6 months has been observed to approach complete recovery following weaning [2,22,25,26,27,28,29,30], some studies have found that lactation past 6 months is associated with only partial recovery [1,16,19,20,31], which suggests that extended lactation can delay the return of BMD to baseline levels. As a result, limited research has focused on the impact of exercise on lactation-related bone loss, with several studies supporting an association between exercise and reduced bone loss [32,33,34] and others reporting no significant difference [17,20,35]. To our knowledge, no studies have used emergent urinary markers of bone resorption such as n-telopeptides (NTX) [36], pyridinoline (PYD), or deoxypyridinoline (DPYD) [37] to help better understand the dynamic changes in bone that occur postpartum.

Changes in serum cholesterol and lipid profiles may also be of concern postpartum. Lipid metabolism adapts during pregnancy to transport adequate cholesterol to the placenta and developing fetus, which supports steroid hormone synthesis and fetal nervous system development. These changes promote maternal fat accumulation during the first two trimesters and enhance the breakdown of fat depots during the third trimester, resulting in hypercholesteremia [8]. While it is well-recognized that cholesterol and TG are elevated during pregnancy due to the aforementioned changes in lipid metabolism, independent of dietary patterns [38], less is known during lactation. The persistence of atherogenic lipid concentrations postpartum has been investigated as a potential predictor of cardiovascular disease risk with research suggesting that lactation may help mitigate elevated plasma cholesterol and TG in postpartum mothers [39,40]. Several studies found that total serum cholesterol and triglyceride concentrations significantly decrease with at least 2 to 6 months of lactation [39,40]. More specifically, a study conducted by Kallio et al. found that total serum cholesterol, LDL-C and HDL-C, and TG all returned to baseline concentrations after one year of exclusive lactation [39]. Limited research has explored the effect of exercise on lipid profiles. For example, a randomized controlled trial by Lovelady et al. [41] found modest increases in HDL-C in women assigned to an aerobic exercise intervention in comparison to sedentary controls but found no changes in other lipid values. Another study that investigated the impact of both diet and exercise on cardiovascular risk factors and weight loss during lactation found no effect of exercise on blood lipids independent of an energy-restricted dietary intervention intended for weight loss [42].

Currently, there is a lack of cohesive research evaluating BMD and/or lipid profile changes across lactation and during postpartum and the potential influence of physical activity, exercise and aerobic fitness during this period. Therefore, the present study aimed to investigate changes in BMD and serum cholesterol and TG across the first postpartum year in lactating women relative to age-matched never-pregnant controls We specifically tested the hypotheses that (1) BMD would decrease during the first 6 months of lactation, followed by at least a partial rebound after weaning; and (2) that previously elevated serum cholesterol and triglyceride concentrations would significantly decrease by 12 months of lactation; Secondary objectives were to evaluate whether habitual physical activity assessed by questionnaire and aerobic fitness would positively influence changes in BMD and serum lipid profile during the 12 months postpartum and explore the use of urinary NTX, PYD, and DPYD as markers of bone resorption during lactation.

## 2. Materials and Methods

This analysis contains longitudinal data collected as part of a study that evaluated the effect of appetite-regulating hormones on body weight retention in lactating mothers [43] and the presence of appetite-regulation hormones in breast milk [44]. Participants were recruited through flyers posted within the community, university, and doctors’ offices. Eligibility criteria included age over 18, singleton birth, 4–6 weeks postpartum, and intentions to fully breastfeed for one year. Participants were excluded if they smoked, had pregnancy complications (e.g., gestational diabetes, preeclampsia), had preexisting kidney, liver, hormonal, stomach, intestine, lung, heart, or blood disease, were taking prescription or over-the-counter medications or herbal supplements, or had a history of anxiety, depression, disordered eating, alcoholism or substance abuse. Twenty-four healthy primiparous women and 20 never-pregnant controls were initially enrolled and provided written, informed consent. Of these, 18 postpartum women and 16 never-pregnant controls completed the one year follow up and were used in the current analysis.

### 2.1. Laboratory Measurements

Measurements were taken at baseline (4 to 6 weeks postpartum), and at 6 and 12 months postpartum in participants and at corresponding time points in control women. At each visit, maternal anthropometrics and body composition/bone density analysis (by dual energy X-ray absorptiometry scan, DXA) were performed, and blood was collected for analysis of serum lipids. Urine was collected in a subset of postpartum (*n* = 14) and control (*n* = 8) women for analysis of urinary markers of bone resorption Physical activity questionnaires were collected at all time points, and a maximal oxygen uptake test (VO_2max_) to assess aerobic fitness was conducted at 12 months. Study visits were scheduled in the follicular phase of the menstrual cycle (1–9 days after the start of menstruation) for never-pregnant control women and for postpartum women who had resumed their menstrual cycles at the 6 and 12 month visits.

### 2.2. Assessment of Anthropometrics and Bone Mineral Density

Participants were measured without shoes and in light clothing. Height was measured using a stadiometer (Invicta Plastics, Leicester, UK) with weight measured on a digital scale (Tanita, Tokyo, Japan). A Gulick tape measure was used to measure maternal hip and waist circumference. Hip circumference was defined as a horizontal measure taken at the maximum circumference of the buttocks [45]. Waist circumference was defined as the narrowest part of the torso above the umbilicus and below the xiphoid process [45]. Bone density and body composition were assessed by DXA (Lunar Prodigy, GE Healthcare, Fairfield, CT). Participants were placed in the supine position, and instructed to lay still on the X-ray table while the fan-beam scanner made a series of transverse scans from head to toe in (0.6- to 1.0-cm) intervals. Three scans were performed: total body, lumbar spine (L1–L4), and dual femur (hips; femoral neck and trochanter). Scans were analyzed utilizing manufacturer-provided software (encore Software v13.6) and standardized for the evaluation of adults. Standardized t-scores, matched against a healthy 30-year female population were recorded for all sites when calculated by the software.

### 2.3. Serum Lipid Profiles

At each testing point, blood for analysis of serum triglycerides (TG), total cholesterol, high-density lipoprotein cholesterol (HDL-C), low-density lipoprotein cholesterol (LDL-C) and very low-density lipoprotein cholesterol (VLDL-C) was collected into serum gel tubes and centrifuged between 30 and 120 min of draw time and kept refrigerated until analysis. Analysis was performed by a commercial laboratory (Regional West Laboratories, Scottsbluff, NE, USA) using standardized procedures. Specifically, TG, total cholesterol and HDL-C were measured by spectrophotometry and LDL-C and VLDL-C were calculated using the Friedewald equation.

### 2.4. Markers of Bone Resorption

Urine samples were collected in the morning (first-morning void) after an overnight fast for analysis of markers of bone resorption including NTX, a general marker of bone-resorption, PYD, a general marker of collagen degradation, and DPYD, a sensitive and specific marker of bone-specific collagen degradation/bone resorption [37] in a subset of postpartum (*n* = 14) and control (*n* = 8) women. Urine samples were immediately frozen (without preservatives) and later analyzed for the aforementioned markers using chemiluminescent immunoassay (NTX, Mayo Clinic Laboratories, Rochester, MN, USA) and high-performance liquid chromatography (PYD, DPYD, ARUP Laboratories, Salt Lake City, UT, USA) by a commercial laboratory (Regional West, Scottsbluff, NE, Powered by Mayo Clinic Laboratories). Values were normalized to urinary creatinine to account for the variation in urinary concentrations between individuals. Urinary creatinine concentration was analyzed via enzymatic colorimetric assay (Mayo Clinic Laboratories, Rochester, MN, USA).

### 2.5. Assessment of Habitual Exercise and Aerobic Fitness

Habitual PA was assessed using the Baecke Physical Activity Questionnaire at each time point. The Baecke questionnaire is a validated assessment tool that categorizes PA into sport, work, and leisure-time activity, and has been shown to be a reliable predictor of habitual PA [46]. Cardiovascular fitness was determined during a walking VO_2max_ test on the treadmill as previously described [43]. Briefly, after a 2 min warm up, the test began with participants walking at 1.1 m/s at 0% grade for one minute. Speed and/or grade was increased each minute in increments appropriate for non-athletic subjects of varying fitness levels to achieve maximal effort within 12–15 min. Heart rate (HR) and rating of perceived exertion (RPE; modified Borg scale) were recorded at the end of the third, sixth, and ninth stages, and at every stage following the ninth. The test was terminated when the participant reached volitional exhaustion. Cardiorespiratory data were collected at 20 s intervals using a computerized system (PARVO, Sandy, UT, USA), O_2_ and CO_2_ analyzers, and a 5 L mixing chamber. To determine if VO_2max_ had been attained, at least two of the following criteria had to be satisfied; Plateau in VO_2_, HR within 10 beats of age-predicted max HR (208 − (0.07 × age)) [47], or respiratory exchange ratio (RER) greater than 1.1 and RPE of ≥ 18.

### 2.6. Menstrual and Lactation Logs

Participants were instructed to keep records of menstrual cycles and lactation frequency throughout the study. Postpartum women were encouraged to breastfeed for a minimum of one year; however, the frequency and duration of lactation were recorded using lactation logs. Menstrual cycle logs were used to determine a return of menses at 6 and 12 months. This information was used to facilitate scheduling of follow-up visits during the follicular phase. Lactation duration and menstrual cycle status were used as group variables in statistical analysis as explained below.

### 2.7. Statistical Analysis

Data were analyzed using SPSS software (Version 26; SPSS Inc., Chicago, IL, USA). Values are reported as mean ± SEM plus data range for all variables except those determined to be highly skewed (skewness <−1 or >1); skewed variables are reported using the median with data range. Differences between body mass, body composition, bone mineral density, serum lipids, and habitual PA between lactating and never pregnant controls were analyzed using independent sample *t*-tests. Changes in BMD, urinary bone resorption markers (subset), and serum lipids across time were evaluated by repeated-measures ANOVA to test for time (baseline, 6 month, and 12 month) and time X group (postpartum vs. control) effects for these and other key variables including PA during the 12-month study period. Paired *t*-tests corrected for multiple comparisons were used to determine differences between baseline and 6 months and baseline and 12 months when a significant time effect was observed. PA and aerobic fitness were added to ANOVA models of postpartum women as a cofactor for variables that changed over time. Independent sample *t*-tests were used to evaluate change in key variables from baseline to 6 months and baseline to 12 months in postpartum women who continued to lactate compared to those who discontinued lactation and to compare women who had a return of their menstrual cycle by 6 months compared to those who did not. Associations between habitual PA and VO_2max_ and the change in BMD and urinary markers were evaluated using Pearson product moment correlations. Statistical significance was set at *p* ≤ 0.05 unless otherwise specified.

## 3. Results

### 3.1. Missing Data

At baseline, hip and waist circumferences were missed for one lactating participant due to recording error. Blood for analysis of lipid profile was missed in 1 control and 2 lactating participants. All data at the six-month collection were missed for one control participant with a scheduling conflict. VO_2max_ data at 12 months could not be obtained from 2 postpartum participants, 1 due to being diagnosed with ataxia oculomotor apraxia type II and one due to computer malfunction. Complete total body t-scores were missing for 2 control and 4 lactating women, complete dual femur t-scores were missing for 4 lactating and control women, and complete lumbar spine t-scores were unavailable for 4 control and 7 lactating women. Incomplete t-scores were present in control and postpartum women who were <21 years old, absent due to a scheduling conflict, or when t-scores were not computed by DXA software.

### 3.2. Body Mass and Body Composition

The 18 postpartum women who completed the study reported weighing 63.7 ± 1.9 kg before pregnancy, gaining 16.0 ± 1.1 kg during pregnancy and delivering term babies which weighed 3.3 ± 0.1 kg at birth. The characteristics of body composition for the postpartum and never-pregnant participants at each time point are shown in Table 1.

At baseline, differences were not detected in age or anthropometric variables except for waist circumference and percent body fat, which was higher (*p* < 0.001) in the postpartum group as previously reported [43]. During the 1-year follow-up, lactating women lost 5.2 ± 1.4 kg and experienced reductions in body fat and waist circumference, whereas the control women remained relatively weight stable (Table 1). The majority of loss in weight, waist circumference, and body fat occurred during the first six months postpartum. At 12 months, body composition did not differ between groups (*p* < 0.05).

### 3.3. Bone Mineral Density and Markers of Bone Resorption

#### 3.3.1. BMD at Dual-Femur, Spine and Total Body

**Figure 1:**
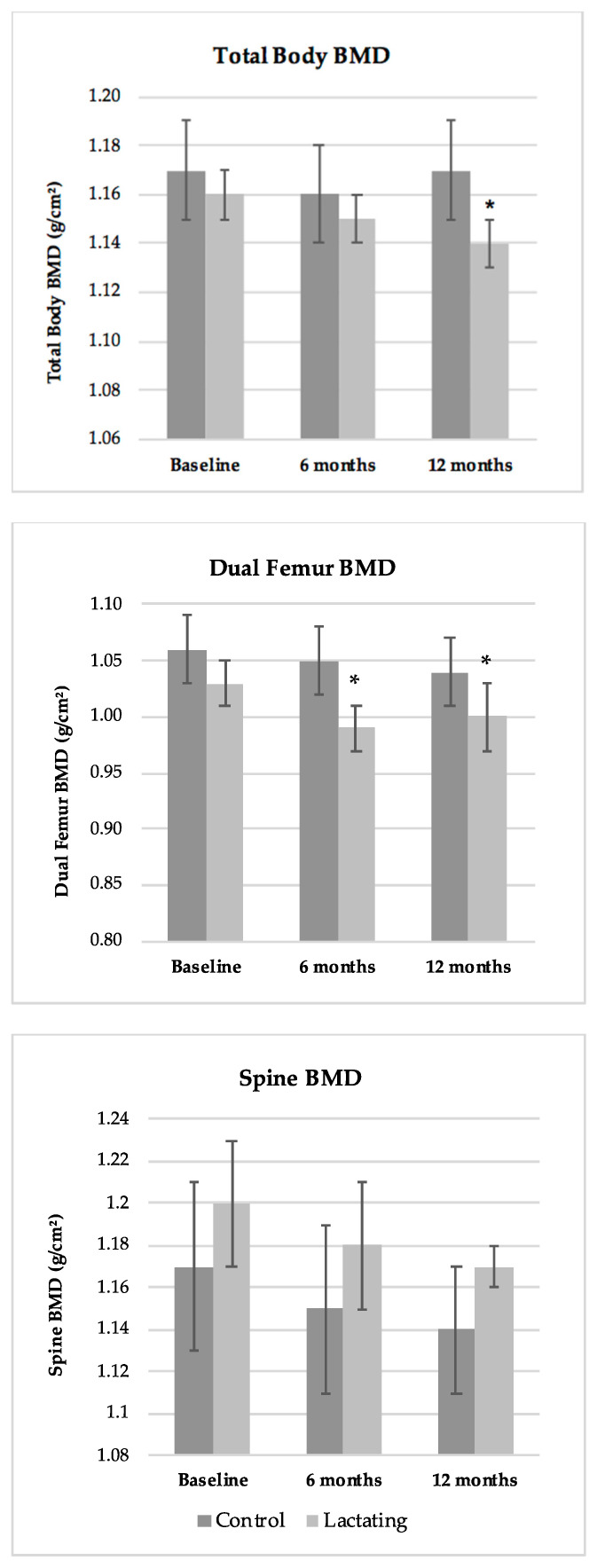
The change in total body (panel A), dual-femur panel B) and lumbar spine (panel C) BMD in lactating verses never-pregnant controls. BMD in total body (time X group interaction, *p* = 0.011) and dual femur (time effect, *p* = 0.014) decreased from 4 to 6 weeks (baseline) to 12 months postpartum. No differences across time were observed for BMD of the spine. * *p* < 0.025 vs. baseline by paired *t*-test.

Bone mineral density at all sites for control and postpartum women is summarized in Table 2 and Figure 1. BMD of total body, dual femur or spine did not differ between postpartum and control women at baseline (*p* = 0.62, *p* = 0.58, and *p* = 0.42, respectively). At baseline, two women in the control group and two in the lactating group had evidence of osteopenia (t-score between −1 and −2.5) in the dual femur, and one of the same controls had evidence of osteopenia in the spine. During the one year follow up, total BMD decreased by 1.4 ± 0.5% and hip BMD decreased by 3.1 ± 0.9% in the postpartum group with changes of +0.2 ± 0.3% and −2.2 ± 1.3% in controls. Although average spine BMD decreased by 2.0 ± 2.3% in the postpartum and 2.2 ± 2.9 in controls (Figure 1), these changes were highly variable among individuals in both groups and did not differ by time or time X group (*p* = 0.19 and *p* = 0.96, respectively).

#### 3.3.2. Biochemical Markers of Bone Resorption

Markers of bone resorption for control and postpartum women are summarized in Table 3. At baseline, urinary PYD and DPYD concentrations were higher in the postpartum compared to the control women (*p* < 0.001) with trends for a higher NTX (*p* = 0.07) and lower DPYD/PYD ratio (*p* < 0.01). All markers changed across time with a significant time X group interaction only for PYD, DPYD, and the PYD/DPYD ratio (Table 3). NTX, PYD and DPYD concentrations at baseline were predictive of greater absolute (data not shown) and percent deficits in dual-femur BMD from baseline to 6 months (r = 0.49, 0.52 and 0.62, respectively, *p* < 0.05) in the full group (*n* = 22) with similar patterns in the postpartum group only (r = 0.41, *p* = 0.15; r = 0.56 and 0.74, *p* < 0.05, respectively).

### 3.4. Serum Lipids

Fasting serum TG and cholesterol concentrations at baseline, 6 months, and 12 months are summarized in Table 4. At baseline, no differences were observed between cholesterol (*p* = 0.42), HDL-C (*p* = 0.33), LDL-C (*p* = 0.12), VLDL-C (*p* = 0.60) or triglyceride concentrations (*p* = 0.57) nor the Cholesterol/HDL-C ratio (*p* = 0.33) between lactating and control women. However, one woman in the control group and 4 in the postpartum group had serum cholesterol concentration of ≥200 mg/dL. Four and 6 control women and 6 and 9 postpartum women had LDL-C and HDL-C concentrations, respectively, that were out of the optimal ranges of <100 mg/dL and >50 mg/dL [48]. During the first postpartum year, women experienced an overall improvement in lipid profile with significant increases in HDL-C concentration and decreases in the cholesterol/HDL-C ratio by 6 months and decreases in LDL-C concentration at 12 months. The control women were observed to have improvements in total and HDL-C during the study period. Changes in VLDL-C and triglyceride concentrations were not observed in either group.

### 3.5. Aerobic Fitness and Physical Activity

Reported habitual PA at baseline, 6 months, and 12 months and VO_2max_ at 12 months are shown in Table 5. Total PA and PA associated with work, sport and leisure-time indices did not differ between lactating and control women at baseline and did not change in either group over time. Therefore, the total Baecke score and the work, sport, and leisure-time indices at all time points were averaged and used for further analysis. All women except one postpartum participant met the criteria for an acceptable VO_2max_ test at 12 months. VO_2max_ was highly variable among control (19.9 to 97.5 percentile) and lactating women (15.1 to 95.7 percentile) [45] but was not different between groups. VO_2max_ was found to correlate with the Baecke sport index (r = 0.523, *p* < 0.01) but not with the work (r = 0.002), leisure-time (r = −0.13) or total Baecke score (r = −0.20) (*p* > 0.05). Results were not affected by the participant who did not have an acceptable VO_2max_ test.

### 3.6. Effect of Habitual Exercise, Lactation and Return of Menses

#### 3.6.1. Habitual exercise and Aerobic Fitness

In postpartum women, measures of PA including VO_2max_, total Baecke score, or work, sport or leisure-time indices did not influence change in BMD across the first postpartum year. The decrease in the Cholesterol/HDL-C ratio was positively influenced by PA during leisure-time (time X group effect (*p* = 0.051, time X group) and sport (*p* = 0.028, time effect). No other interactions were observed.

#### 3.6.2. Lactation Duration

Nine of the 18 postpartum women (50%) continued to breastfeed for the full postpartum year while nine had stopped before (*n* = 1) or shortly after the 6 month (*n* = 8) visit. Those who continued to lactate for 12 months had a greater fall in BMD in the total body (0.0311 ± 0.0092 g/cm^2^) and hip (0.0542 ± 0.0083 g/cm^2^) than those who had stopped lactating (0.0028 ± 0.0048 g/cm^2^ and 0.0117 ± 0.0138 g/cm^2^, respectively (*p* < 0.05)). Differences were not detected in the spine (*p* = 0.638). There was also no evidence of BMD re-bound at 12 months in any site. In the subset with urinary bone resorption markers, those who continued to lactate (*n* = 8) had less of a fall in NTX (4.8 ± 10.6 vs. 57.3 ± 21.6 nM BCE/mM creatinine), PYD (63.5 ± 12.2 vs. 107.4 ± 12.7 µmol/mol creatinine), and DPYD (5.8 ± 2.7 vs. 18.5 ± 3.4 µmol/mol creatinine) than those (*n* = 6) who stopped earlier. There were no differences observed for the change in serum lipid profile.

#### 3.6.3. Return of Menses

Six of the 18 postpartum women (33%) had resumed menses by six months postpartum whereas 12 (67%) resumed menses after six months, with 10 of the 12 not experiencing menses by study end. The change in hip BMD from baseline to 12 months was positively influenced by the return of menses in that those who had a return of menses before 6 months had a dampened fall in BMD in the hip (0.0047 ± 0.0148 g/cm²)) compared to those who didn’t who experienced a greater fall in BMD (0.04709 ± 0.00994 g/cm²) (*p* = 0.028). Resumption of menstrual cycle did not impact other areas of BMD or the change in serum lipid profile.

## 4. Discussion

Physiological changes that occur following pregnancy and during lactation can have an impact on bone health and cardiovascular risk. The current study evaluated the longitudinal changes in BMD and serum lipids and TG across the first postpartum year in lactating women relative to age-matched never pregnant controls. While results confirm previous, somewhat inconsistent findings that observed decreases in BMD during lactation, they further highlight that BMD rebound does not necessarily occur by 12 months in those continuing to breastfeed, and that BMD loss occurs in never-pregnant controls regardless of PA patterns. Results also add to the currently limited studies evaluating serum lipids during postpartum and with lactation that demonstrate general improvements in HDL-C, LDL-C and the cholesterol/HDL-C ratio independent of habitual PA. Additionally notable was our exploratory observation in a subset of participants that urinary biomarkers of bone resorption, namely NTX, DPYD and PYD, may help better understand changes in BMD in the postpartum period. Overall these findings contribute to understanding of whether persistent reductions in BMD and elevated serum lipids postpartum increase risk for osteoporosis and CVD later in life.

Our results specifically demonstrate the impact of breastfeeding on BMD in the dual femur, lumbar spine, and total body, and support the concept that skeletal demineralization and bone resorption during lactation support the calcium demands of milk production [10,49]. We chose to examine total body BMD, which overall contains a high content of cortical bone, as well as the dual femur (hip) and lumbar spine that are rich in trabecular bone. We found that the lactation-induced effect on maternal bone is particularly pronounced in the total body [13,18,24,50,51] and hip [13,16,17,20,22,23,24,35,50,52,53,54]. The calcium demand of producing breast milk, however, should theoretically have a greater influence on trabecular bone because it has a higher turnover rate and is more metabolically active than cortical bone [55]. In a 24-month longitudinal study, Hopkinson and colleagues [18] found that women who breastfed for a longer duration experienced loss in bone mineral accretion in cortical-rich sites but that trabecular-rich sites were the first to recover from lactation-induced BMD loss. Several other well-controlled studies support these findings [1,2,16,23,31].

The current study found that lactating women had an average decrease of 1.4% in total body BMD from early (4–6 months) to 12 months postpartum, which was significantly greater than the average total body BMD loss observed in the control group. The magnitude of these lactation-induced changes in total body BMD is in agreement with previous studies which have reported deficits ranging from 0.86 to 3% within the first 6 months of lactation [2,13,18,19,24,50,53] but not in alignment with others that found no changes in BMD during the first 3 months postpartum [35,49] and post-weaning [53]. Similar to the current study, DXA was employed to measure BMD in the majority of previous studies. However, variations in breastfeeding frequency, duration, time of weaning and sample size may explain the discrepancy in results. To determine the clinical significance of this loss, we further evaluated t-scores, which standardizeresults to those of healthy 30-year-old female adults. Despite the significant reduction across the 12-months postpartum, osteopenia risk (t-score <−1 and >−2.5) [56] was not immediately increased except in the spine of two postpartum participants who had baseline t-scores in the lower range (0.7 and 0.9) that dropped into the osteopenic range during the study (−1.2 and −1.3). The two lactating and two control women who were at risk at baseline based on dual femur t-scores remained at risk.

In addition, our results show an average decrease of 3.1% in dual femur BMD in the postpartum group, which agrees with previous studies that found deficits ranging from 2 to 7% [2,3,13,17,20,35,52,53,54]. Unlike BMD of total body, the decrease in dual femur BMD occurred in both groups over the year of study follow up with the controls experiencing an average decrease of 2.1%. These results align with existing research in postpartum women that found initial recovery to originate in trabecular-rich sites, such as the dual femur, by 12 months [1,2,16,18,23,31]. Contrary to the majority of previous studies, we did not find a significant decline in spine BMD [13,16,18,20,22,23,24,35,49,50,51,52,54,57,58], which may be due to notable variation among individual women in both the postpartum and control groups. There is also the possibility early postpartum changes in spine BMD (i.e., over the first 4 to 6 weeks), before our baseline assessment, were missed. By comparison, bone loss occurs at an annual rate of −1.8 to −2.5% in lumbar spine and −1.0 to −1.7% in the hip in the perimenopausal period [59] and may be as high as −3.3% in the spine and −2.0% in the femoral neck following menopause [60].

Although there was a slight average increase (1.01%) in dual-femur BMD during the 6- to 12-month period, this change was not statistically significant and could not be considered a rebound of BMD, as has been reported by many previous studies [61]. According to a systematic review by Grizzo et al. [61], the majority of BMD loss occurs during the first 6 months of lactation with several studies demonstrating complete or almost complete recovery to baseline levels at all skeletal sites measured post-weaning. However, other studies only showed partial recovery to pre-pregnancy BMD levels, especially in those who lactated for more than 6 months [1,16,18,19,20,31]. A well-controlled study by Cross et al. [49] found that spinal BMD lost during lactation was regained approximately 3 months after weaning, regardless of the duration of breastfeeding. Other research suggests that recovery of BMD is site specific and takes as long as 6 to 7 months post-weaning for sites such as the hip [18,62]. Given that 50 percent of our participants were still lactating at 12 months, a rebound in BMD by study end may not be expected. The mechanism of bone recovery after lactation is unknown, but reestablishment of ovarian hormones leading to resumption of ovulation and decreased prolactin (PRL) production following weaning may influence recovery [63]. Not surprisingly, the fall in BMD from baseline to 12 months in the total body and dual femur was greater in those who continued to breastfeed for a full year compared to those who stopped lactating earlier, which at least in the hip was related to the return of menstrual function and estrogen cycling in women who ceased breastfeeding. Additionally, we found no evidence supporting a beneficial effect of habitual PA (either from sport, work or leisure-time) or aerobic fitness on change in postpartum BMD or the propensity for BMD rebound. From previous studies, a beneficial effect of physical activity is inconsistently observed. Previous observational studies by Little et al. [35] and Sowers et al. [20] reported no association between exercise and dampened BMD loss over 12 months in lactating women who participated in self-selected exercise. In contrast, two more recent randomized controlled trials found that those assigned to an exercise intervention group (which included both resistance and aerobic exercise training) experienced less BMD loss in the lumbar spine, but not the total body or hip, than those in the control group [32,33].

A secondary purpose of the current study was to evaluate the usefulness of urinary biochemical markers of bone resorpton in postpartum, lactating women. We showed, in a subset of participants, that all urinary markers including NTX, PYD and DPYD decreased from early to 12 months postpartum. NTX is a urinary amino-terminal cross-linking telopeptide of type I collagen whereas urinary DPYD is derived from proteolytic hydrolysis of collagen found in bone, and PYD is a less specific marker of bone as well as cartilage, tendon, and blood vessels [37]. As these markers are byproducts of bone remodeling that can be detected in urine, the reduced urinary concentrations across ~one to six and 12 months postpartum is indicative of decreased bone resorption across this same time period. The increase in the DPYD/PYD ratio, however, suggests that a proportion of this reduced resorption may be from collagen that is not necessarily specific to bone. Additionally, our finding that baseline NTX, PYD, and DPYD concentrations were predictive of changes in dual-femur BMD during the first six months postpartum suggest that these markers could be useful in future studies of pregnant and postpartum women, or at other times when younger women may be at risk for increased bone resorption. Urinary markers are thought to be a more cost-effective and obtainable measures of BMD and are more reflective of current bone status as compared to DXA, which provides only a static snapshot of BMD [36]. Thus, in clinical settings like the current, where BMD values show no observable signs of rebound, urinary markers may be useful to further explore the changes in skeletal demineralization and bone resorption in association with weaning and/or the return of menses that occur before observed changes in BMD. Use of these markers, however, need to be further evaluated for sensitivity and specificity as not all findings in our control women, including the fall in NTX concentration in light of stable PYD and DPYD, can be easily explained.

A second major objective of this study was to investigate changes in cholesterol and lipid profiles in the year following childbirth and throughout lactation. While hypercholesterolemia is common during gestation due to changes in lipid metabolism [8], a persistent elevation of atherogenic lipid concentrations postpartum may increase cardiovascular disease risk with research suggesting that lactation may help mitigate elevated plasma cholesterol and TG in postpartum mothers [39,40,64]. Little is known about the effect of postpartum exercise. Our results are in partial support of both our initial hypothesis and limited previous research that found a reduction in both total serum cholesterol and LDL-C following at least two to six months of lactation [10,40,65]. While we observed decreases of −8.4% and −15.7% in total serum cholesterol and LDL-C concentrations, respectively, in the postpartum group, only the drop in LDL-C was significant and not until 12 months postpartum. The changes in total cholesterol were highly variable among both postpartum and control women, as were the changes in TG which did not change throughout the study in either group. This is in contrast with existing research that demonstrated decreases in TG during lactation [10,40,65] and could be attributed to the presence of confounding variables in both the postpartum and control groups which include variations in dietary intake, body weight and alcohol consumption. The current study, however, did observe a significant improvement in HDL-C and cholesterol/HDL-C ratio which was evident by 6 months. There is limited research surrounding the effects of exercise on expediting the return of cholesterol and TG to baseline levels. An earlier study by Lovelady et al. [41] found a slight increase in HDL-C in lactating women assigned to an exercise intervention when compared to the non-exercising control group. Our data support this, indicating that both Leisure-time and Sport PA Indices were influential on the change in the cholesterol/HDL-C ratio over the first postpartum year.

Although our study provides insight into the changes in BMD and serum lipid profile during the first postpartum year, it is limited by a small sample size, absence of pre-pregnancy data and a non-lactating postpartum group, time frame of follow up, and methodological concerns of assessing PA by self-report questionnaire. The majority of previous studies utilized sample sizes between 6 and 139 lactating women and between 9 and 98 controls. Our sample size was on the lower end, which did not allow us to simultaneously account for all potentially important cofounders (lactation duration, menstrual cycle status, seasonality, etc.) in our repeated measures analysis models. Additionally, the inclusion of a non-lactating postpartum group would have allowed for teasing out the effect of lactation vs. childbearing on BMD and serum lipids. Unfortunately, recruitment of a non-breastfeeding control group proved impossible because of the high breastfeeding initiation rates of our state (>80%) (https://www.cdc.gov/breastfeeding/data/reportcard.htm accessed on 24 January 2022). Most importantly, however, a longer duration of follow-up which included testing pre-pregnancy, in earlier postpartum (several days after delivery), and as far out as 18 or 24 months postpartum would have allowed us to better capture true changes in BMD and serum lipids postpartum and determine the pattern of these changes and presence of a true rebound to pre-pregnancy values. Finally, our assessment of PA was limited because it addressed PA in general, albeit from sport, work and leisure-time, and did not specifically address exercise intensity or differentiate between weight bearing vs. non-weight bearing activity. Questionnaires, such as the Baecke PA Questionnaire, can be subject to recall and social bias and while VO_2max_ is commonly used to assess cardiovascular fitness, it is still influenced by genetics and does not account for how exercise habits may have changed after giving birth. It is also important to consider that our participant population consisted of relatively healthy and active women living in a university town, which may have reduced the amount of variation necessary to detect a difference and limit the generality of our findings.

## 5. Conclusions

Results of the present study confirm previous investigations which observed decreases in BMD during lactation and highlight that BMD rebound does not necessarily occur by 12 months in women who continue to breastfeed into the latter part of the first postpartum year. They, however, provide evidence that the overall lipid profile improves from early to late postpartum, with significant increases in HDL-C concentration and decreases in the cholesterol/HDL-C ratio by 6 months and decreases in LDL-C concentration at 12 months. Breastfeeding status was observed to influence the change in BMD in the dual femur and total body, whereas reported physical activity during sport and leisure heightened the change in the Cholesterol/HDL ratio. The potential benefits of exercise and use of urinary biomarkers of bone deserves further exploration during and following pregnancy and lactation.

## Figures and Tables

**Table 1:** Anthropometric Characteristics in Lactating (*n* = 18) and Control (*n* = 16) Women.

		Baseline	6 Months	12 Months	*p* Value
Age (years)	Control	26.4 ± 1.4	-	-	-
		(19–38)			
	Lactating	27.9 ± 1.5	-	-	
		(19–38)			
Height (cm)	Control	169.0 ± 1.4	-	-	-
		(160.0–178.5)			
	Lactating	167.7 ± 1.8 (154.1–179.2)	-	-	
Weight (kg)	Control	68.0 ± 2.2	69.1 ± 2.3	67.9 ± 2.5	* *p* = 0.019
		(55.4–86.1)	(55.6–87.4) ^a^	(54.2–92.2)	
	Lactating	70.8 ± 2.2	66.8 ± 2.3	65.6 ± 2.3	
		(52.8–92.3)	(48.5–85.2) ^1^	(48.2–86.6) ^2^	
BMI (kg/m²)	Control	23.9 ± 0.7	24.2 ± 0.7	23.7 ± 0.8	* *p* = 0.026
		(19.5–30.0)	(20.3–30.2) ^a^	(19.0–32.2)	
	Lactating	25.0 ± 0.8	23.6 ± 0.9	23.0 ± 0.9	
		(20.3–32.9)	(19.0–32.0) ^1^	(18.4–33.1) ^2^	
Waist Circumference (cm)	Control	81.5	81.1	80.3	* *p* = 0.027
		(68.0–101.0)	(72.5–104.0) ^a^	(29.5–106.0)	
	Lactating	93.5	83.8	80.5	
		(77.0–107.0) ^b^	(32.0–109.4)	(71.5–105.4) ^2^	
Hip Circumference (cm)	Control	99.0 ± 1.9	102.3 ± 1.4	97.7 ± 4.4	* † NS
		(84.5–110.0)	(92.7–110.6) ^a^	(37.5–115.0)	
	Lactating	104.5	98.8	99	
		(85.5–118.0) ^b^	(37.5–117.2)	(86.6–120.7)	
Body Fat (%)	Control	34.2 ± 1.8	36.0 ± 1.5	34.5 ± 2.0	* *p* = 0.007
		(18.0–44.8)	(26.1–44.1) ^a^	(15.4–46.4)	
	Lactating	38.9 ± 1.4	36.0 ± 1.7	34.5 ± 1.9	
		(26.5–47.0)	(24.4–52.3) ^1^	(22.5–52.9) ^2^	

Results reported as mean ± SEM (range); ^a^ missing data due to scheduling conflict (*n* = 1) or ^b^ recording error (*n* = 1); * significant time X group interaction by repeated measures ANOVA; † significant time effect by repeated measures ANOVA; ^1^ significant difference by paired *t*-test 6 months vs. baseline (*p* < 0.025); ^2^ significant difference by paired *t*-test 12 months vs. baseline (*p* < 0.025); NS = no significant time or time X group effect by repeated measures ANOVA.

**Table 2:** Bone Mineral Density in Lactating (*n* = 18) and Control (*n* = 16) Women.

Bone Density		Baseline	6 Months	12 Months	*p* Value
Total Body (g/cm²)	Control	1.17 ± 0.02	1.16 ± 0.02	1.17 ± 0.02	* *p* = 0.011
		(1.07–1.27)	(1.06–1.27) ^a^	(1.07–1.28)	
	Lactating	1.16 ± 0.01	1.15 ± 0.01	1.14 ± 0.01	
		(1.08–1.31)	(1.07–1.24)	(1.04–1.23) ^2^	
Spine (g/cm²)	Control	1.17 ± 0.04	1.15 ± 0.04	1.14 ± 0.03	* † NS
		(0.94–1.41)	(0.82–1.39) ^a^	(0.93–1.40)	
	Lactating	1.20 ± 0.03	1.18 ± 0.03	1.17 ± 0.01	
		(0.99–1.34)	(0.96–1.46)	(0.81–1.40)	
Dual Femur (g/cm²)	Control	1.06 ± 0.03	1.05 ± 0.03	1.04 ± 0.03	† *p* = 0.014
		(0.85–1.33)	(0.84–1.24) ^a^	(0.84–1.19)	
	Lactating	1.03 ± 0.02	0.99 ± 0.02	1.00 ± 0.03	
		(0.87–1.21)	(0.82–1.13) ^1^	(0.85–1.15) ^2^	
T-scores Total Body	Control	0.59 ± 0.19	0.56 ± 0.21	0.54 ± 0.20	† *p* = 0.041
		(−0.5 – 1.7) ^c^	(−0.8 – 1.8) ^ac^	(−0.7 – 1.9)	
	Lactating	0.49 ± 0.17	0.29 ± 0.16	0.22 ± 0.13	
		(−0.5 – 2.3) ^d^	(−0.7 – 1.4) ^bc1^	(−0.6 – 1.3)	
T-scores Spine	Control	0.41 ± 0.28	0.28 ± 0.30	0.31 ± 0.32	* † NS
		(−2.1 – 1.8) ^c^	(−2.0 – 1.6) ^ad^	(−2.2 – 2.3) ^d^	
	Lactating	0.35 ± 0.17	0.16 ± 0.26	0.18 ± 0.22	
		(−0.9 – 1.2) ^e^	(−1.2 – 2.1) ^be1^	(−1.7 – 1.7) ^c^	
T-scores Dual Femur	Control	0.45 ± 0.23	0.29 ± 0.26	0.06 ± 0.22	† *p* = 0.009
		(−1.3 – 1.7) ^c^	(−1.4 – 1.8) ^ad^	(−1.3 – 1.4) ^d^	
	Lactating	0.25 ± 0.20	−0.18 ± 0.17	−0.12 ± 0.17	
		(−1.1 – 1.6) ^d^	(−1.2 – 1.0) ^bc1^	(−1.1 – 1.1)^2^	

Results reported as mean ± SEM (range); ^a^ missing data due to scheduling conflict (*n* = 1) or ^b^ (*n* = 2); ^c^ missing t-score (*n* = 1) or ^d^ (*n* = 2) or ^e^ (*n* = 3); * significant time X group interaction by repeated measures ANOVA; † significant time effect by repeated measures ANOVA; ^1^ significant difference by paired *t*-test 6 months vs. baseline (*p* < 0.025); ^2^ significant difference by paired *t*-test 12 months vs. baseline (*p* < 0.025); NS = not significant.

**Table 3:** Urinary Biochemical Markers of Bone Resorption in a Subset of Lactating (*n* = 8) and Control (*n* = 5) Women.

		Baseline	6 Months	12 Months	*p* Value
NTX (nM BCE/mM creatinine)	Control	67.1 ± 11.1	54.3 ± 6.7	46.6 ± 3.4	† *p* = 0.007
		(25–121)	(26–76)	(31–57)	
	Lactating	101.1.6 ± 14.0	101.1 ± 8.0	73.8 ± 7.0	
		(34–219)	(66–156)	(28–124)^2^	
PYD (µmol/mol creatinine)	Control	46.5 ± 4.5	47.7 ± 3.7	50.7 ± 3.7	* *p* = 0.000
		(34.3–72.8)	(34.6–64.4)	(38–69.6)	
	Lactating	151.3 ± 11.8	80.4 ± 5.5	69.0 ± 4.4	
		(101.5–266.7)	(59.4–119.9) ^1^	(43.4–100.7) ^2^	
DPYD (µmol/mol creatinine)	Control	13.9 ± 1.4	14.4 ± 1.4	14.8 ± 1.3	* *p* = 0.021
		(8.8–20.9)	(8.4–18.9)	(9.0–20.6)	
	Lactating	34.3 ± 2.7	26.9 ± 2.0	23.1 ± 1.7	
		(23.2–64.4)	(18.4–37.8) ^1^	(14.2–32.7) ^2^	
DPYD/PYD Ratio	Control	0.31 ± 0.01	0.31 ± 0.01	0.29 ± 0.01	* *p* = 0.000
		(0.25–0.42)	(0.23–0.34)	(0.24–0.32)	
	Lactating	0.23 ± 0.01	0.33 ± 0.01	0.33 ± 0.01	
		(0.17–0.36)	(0.25–0.44) ^1^	(0.27–0.42) ^2^	

NTX, N-telopeptide PYD, pyridinoline; DPYD, deoxypyridinoline. Results reported as mean ± SEM (range); * significant time X group interaction by repeated measures ANOVA; † significant time effect by repeated measures ANOVA; ^1^ significant difference by paired *t*-test 6 months vs. baseline (*p* < 0.025); ^2^ significant difference by paired *t*-test 12 months vs. baseline (*p* < 0.025); NS = no significant time or group effect by repeated measures ANOVA.

**Table 4:** Cholesterol and Triglyceride (TG) Concentrations in Lactating (*n* = 18) and Control (*n* = 16) Women.

		Baseline	6 Months	12 Months	*p* Value
Cholesterol (mg/dL)	Control	163.1 ± 6.5	186.3 ± 7.5	183.8 ± 8.4	* *p* = 0.002
		(108.0–208.0) ^b^	(154.0–253.0) ^a1^	(133.0–270.0) ^2^	
	Lactating	171.6 ± 8.0	165.6 ± 5.8	157.2 ± 5.7	
		(115.0–227.0) ^b^	(130.0–204.0)	(113.0–206.0)	
HDL-C (mg/dL)	Control	51.1 ± 3.6	62.3 ± 4.7	59.3 ± 3.4	† *p* = 0.000
		(32.0–73.0) ^b^	(37.0–109.0) ^a1^	(32.0–89.0) ^2^	
	Lactating	46.7 ± 2.6	53.7 ± 2.3	52.3 ± 2.1	
		(29.0–68.0) ^b^	(31.0–68.0) ^1^	(39.0–71.0)	
LDL-C (mg/dL)	Control	91.7 ± 6.3	103.2 ± 6.6	104.3 ± 9.3	* *p* = 0.010
		(53–156) ^b^	(67.0–170.0) ^a^	(44.0–212.0)	
	Lactating	106.4 ± 6.8	95.6 ± 4.8	89.7 ± 4.1	
		(70.0–165.0) ^b^	(69.0–124.0)	(58–122) ^2^	
VLDL-C (mg/dL)	Control	19	21	19	* † NS
		(10.0–49.0) ^b^	(9.0–36.0) ^a^	(10.0–45.0)	
	Lactating	18.6 ± 2.5	16.4 ± 1.8	15.2 ± 2.0	
		(10–47) ^b^	(9–39)	(9–47)	
TG (mg/dL)	Control	96	103	93.5	* † NS
		(52.0–244.0) ^b^	(46.0–179.0) ^a^	(51.0–227.0)	
	Lactating	76.1	71	64.5	
		(50–234) ^b^	(43–195)	(47–234)	
Cholesterol/HDL-C Ratio	Control	3.1	2.8	2.9	† p = 0.001
		(2.1–6.5) ^b^	(2.1–6.0) ^a1^	2.0–7.1)	
	Lactating	3.5	3	3	
		(2.7–6.7) ^b^	(2.4–6.0) ^1^	(2.4–3.8) ^2^	

Results reported as mean ± SEM (range); ^a^ missing data due to scheduling conflict; ^b^ missing blood sample collection (*n* = 1 control and 2 lactating); * significant time X group interaction by repeated measures ANOVA; † significant time effect by repeated measures ANOVA; ^1^ significant difference by paired *t*-test 6 months vs. baseline (*p* < 0.025); ^2^ significant difference by paired *t*-test 12 months vs. baseline (*p* < 0.025); NS = no significant time or time X group effect by repeated measures ANOVA.

**Table 5:** Reported Physical Activity and Aerobic Fitness in Lactating (*n* = 18) and Control (*n* = 16) Women.

		Baseline	6 Months	12 Months	*p* Value
Work Index	Control	2.5 ± 0.1 (1.6–3.5)	2.4 ± 0.1 (1.6–3.1) ^a^	2.6 ± 0.2 (1.4–3.9)	* † NS
	Lactating	2.2 ± 0.2 (0.0–3.1)	2.3 ± 0.1 (1.6–3.3)	2.3 ± 0.1 (1.6–3.3)	
Sport Index	Control	3.7 ± 0.2 (2.3–4.5)	3.2 ± 0.2 (2.0–4.8) ^a^	3.4 ± 0.2 (2.0–4.5)	* † NS
	Lactating	2.4 ± 0.2 (1.0–3.5)	2.5 ± 0.2 (1.3–3.8)	2.7 ± 0.2 (1.5–4.5)	
Leisure Index	Control	3.6 ± 0.4 (2.5–10.0)	3.7 ± 0.3 (2.3–7.3) ^a^	3.6 ± 0.4 (2.8–8.9)	* † NS
	Lactating	2.9 ± 0.2 (1.5–6.5)	2.8 ± 0.1 (2.0–3.5)	2.8 ± 0.1 (2.0–3.8)	
Total Baecke Score	Control	9.8 ± 2.2 (7.3–16.8)	9.3 ± 1.6 (6.5–12.5.0) ^a^	9.6 ± 2.2 (6.1–15.6)	* † NS
	Lactating	7.5 ± 0.4 (3.5–11.3)	7.6 ± 0.2 (5.4–10.3)	7.8 ± 0.3 (5.9–10.0)	
VO_2max_ (ml/kg/min)	Control	-	-	37.9 ± 1.7 (24.2–50.9)	-
	Lactating	-	-	37.5 ± 1.5 (29.1–48.7) ^b^	

Results reported as mean ± SEM (range); ^a^ missing data due scheduling conflict (*n* = 1); ^b^ unable to complete aerobic fitness test = 2); * = significant time X group interaction by repeated measures ANOVA; † = significant time effect by repeated measures ANOVA; NS = no significant time or time X group effect by repeated measures ANOVA.

## Data Availability

The data presented in this study are available on reasonable request from the corresponding author. The data are not publicly available due to privacy concerns.
